# Intelligibility of first-language (L1) and second-language (L2) speech by switched-dominance Spanish-English bilinguals

**DOI:** 10.1121/10.0003688

**Published:** 2021-03

**Authors:** Michael Blasingame, Ann R. Bradlow

**Affiliations:** Department of Linguistics, Northwestern University, 2016 Sheridan Road, Evanston, Illinois 60208, USA mblasingame@u.northwestern.edu, abradlow@northwestern.edu

## Abstract

Recordings of Spanish and English sentences by switched-dominance bilingual (SDB) Spanish (i.e., L2-dominant Spanish-English bilinguals) and by L1-dominant Spanish and English controls were presented to L1-dominant Spanish and English listeners, respectively. At –4 dB signal-to-noise ratio (SNR), Spanish and English productions by SDBs were equally intelligible with both reaching L1-dominant control levels. At –8 dB SNR, SDB English intelligibility matched that of L1-dominant English controls, yet SDB Spanish intelligibility was significantly lower than that of L1-dominant Spanish controls. These results emphasize that extended (but not early) exposure is both necessary and sufficient for robust speech learning.

## 1. Introduction

Language learning is shaped by both timing (i.e., age) and amount (i.e., duration) of linguistic exposure. While by definition typical late bilingual and monolingual populations confound these two factors—earlier onset typically goes with longer duration of acquisition resulting in first-language (L1) dominance—switched-dominance bilinguals (SDBs) dissociate them. With early language exposure being limited to the “home” language (the L1), these bilinguals ultimately have more extensive exposure to the language that predominates in the society-at-large resulting in a switch from L1 to L2 dominance, where language dominance is defined as the language which is used most often.

Prior research on speech production by SDBs has demonstrated some benefit of early exposure on speech sound production in the non-dominant L1 relative to late learners of the language [for current research, see the recent special issue of Languages: Contemporary Advances in Linguistic Research on Heritage Spanish Hoot (2020)]. For example, Au *et al.* (2002) found that despite clear dominance in the L2 (English), SDB Spanish speakers (L1 non-dominant Spanish, L2 dominant English) were still able to produce native-like voice onset times (VOTs) (pre-voiced versus short lag) for phonological voicing contrasts in Spanish. Talkers in an L2 learner comparison group (L1-dominant English; acquired Spanish after age 14), however, produced more English-like VOTs for Spanish stop initial words (short lag versus long lag/aspiration). Oh *et al.* (2003) showed similar maintenance of the Korean three-way laryngeal contrast system for SDB Korean speakers (L2-dominant English). Additionally, Chang *et al.* (2011) demonstrated production of acoustically distinct speech categories for Mandarin versus English back vowels by SDB Mandarin speakers (L2-dominant English, L1 non-dominant Mandarin).

In these prior studies [e.g., Au *et al.* (2002), Chang *et al.* (2011), and Oh *et al.* (2003)], the focus was on individual segment productions. A general finding of studies of connected speech recognition for both L1 and L2 speech is that variation in intelligibility depends on a plethora of acoustic dimensions [see Uchanski (2005), Smiljanic and Bradlow (2009), and Smiljanic (2021) for extensive discussions of the acoustic correlates of variation in speech intelligibility]. Furthermore, objective intelligibility as assessed by proportion of words correctly recognized is distinct from subjective judgements of comprehensibility or “accentedness” [e.g., Derwing and Munro (1997) and Munro and Derwing (1995)]. Thus, in order to more fully understand the effects of early and extended exposure on speech production in each of the languages of SDBs, the present study examined connected speech intelligibility beyond phonetic realization of individual segments (i.e., word recognition accuracy in sentence contexts).

In a companion study to the present study of intelligibility of speech produced by SDBs, Blasingame and Bradlow (2020) focused on SDBs' recognition accuracy of speech produced by L1 speakers in each of their languages (i.e., on switch dominance bilinguals' speech perception). This prior study compared recognition of words in sentence-final position by SDB Spanish speakers to L1-English/L2-Spanish bilinguals and L1-Spanish/L2-English bilinguals (i.e., L1-dominant, late L2 non-dominant learners) under a variety of signal enhancements and degradations. The stimuli for these speech recognition comparisons were produced by L1 speakers of both English and Spanish. This study found that in favorable listening conditions (e.g., low levels of background noise), SDB had L1-like levels of speech recognition accuracy (compared to the L1-dominant control listeners) in both languages, consistent with the claim of Au *et al.* (2002) and Oh *et al.* (2003) that early exposure to the non-dominant language provides some persistent benefits to speech learning. However, only the dominant L2 of the SDB (here, English) showed L1-like resistance to the presence of noise as measured by sentence-final word recognition accuracy in unfavorable listening conditions. This finding suggests that later but extended exposure to English was sufficient for SDB to achieve L1-like speech recognition accuracy in their later-acquired but now-dominant L2 (English). In contrast, early but limited exposure to Spanish was not sufficient to ensure typical L1-like resistance to high levels of noise for Spanish speech-in-noise recognition.

Thus, while the segment production evidence [e.g., Au *et al.* (2002), Oh *et al.* (2003), and Chang *et al.* (2011)] suggests a lasting influence of *early* exposure on non-dominant L1 production by SDB speakers, the speech-in-noise recognition accuracy evidence [e.g., Blasingame and Bradlow (2020)] suggests that *extended* exposure is necessary for robust speech recognition accuracy in the non-dominant L1. Given these influences of both early exposure and extended exposure on speech learning, we ask whether the benefits of early exposure for production of phoneme contrasts by SDBs [i.e., as demonstrated for VOT and vowel contrast production in Au *et al.* (2002) and Oh *et al.* (2003)] generalizes to connected speech production by SDBs in their non-dominant L1 as compared to their dominant L2. Furthermore, we ask if the observed vulnerability to disruption of non-dominant L1 speech recognition accuracy by SDBs under highly unfavorable listening conditions also emerges for their non-dominant L1 overall speech intelligibility. Specifically, we examine the intelligibility of words in sentences produced by SDBs in both their non-dominant L1 and their dominant L2 for L1 listeners of each language under both favorable and unfavorable listening conditions.

## 2. Methods and materials

### 2.1 Talkers

All talkers and listeners filled out a written language survey regarding their demographic information and language use as reported below. SDB (n = 11) were recruited via fliers on Northwestern University's campus. Their ages ranged from 18 to 22 years old. All SDB reported acquiring Spanish at birth (age 0), ensuring that Spanish was their first-acquired language (from here L1 will refer to first-acquired language and L2 will refer to second-acquired language), and English between ages 5 and 8 years old. All 11 SDB participants were born in the U.S. Spanish usage at home was reported as exclusive (100%) during early childhood (before age 5) but falling to less than 20% during adulthood. Conversely, English usage during early childhood was nearly non-existent (less than 30% for all participants), yet between 80% and 100% usage during adulthood. Critically, SDB took no courses in high school in which the medium of instruction was any other language besides English, ensuring language dominance in English based on their frequency of English usage. Further support comes from a written cloze test given to all SDB as an additional measure of language proficiency. The average SDB English score was 37.1 out of 40 (s.d. 1.85) and SDB Spanish score was 35 out of 40 (s.d. 1.74).

English controls (n = 11) were recruited via the Northwestern University Linguistics department subject pool. Their ages ranged from 18 to 22 years old. All English controls reported acquiring English at birth (age 0) and using English exclusively in the home during childhood. Usage of English at home and in society was reported at 100% during both childhood and adulthood. Critically, English controls took no courses in high school in which the medium of instruction was any other language besides English, ensuring that English is both their L1 and dominant language. English control participants with second-language education (e.g., Spanish courses in high school) were included although no English control reported acquiring a second language before age 8.

Spanish controls (n = 11) were recruited via fliers on Northwestern University's campus. Participants were all graduate students at Northwestern University. Their ages ranged from 23 to 35 years old. All Spanish controls reported acquiring Spanish at birth (age 0) and using Spanish exclusively in the home during childhood. Spanish controls were born in Mexico (n = 5), Chile (n = 3), Ecuador (n = 2), and Peru (n = 1). At the time of the study, Spanish controls used English rather extensively in their graduate school careers (ranging from 20 percent to 80 percent English usage at Northwestern University) compared to their exclusive Spanish usage during childhood (near 100% Spanish usage) and before arrival in the USA for graduate school. Critically, Spanish controls took no courses in high school in which the medium of instruction was any other language besides Spanish, ensuring that Spanish is both their L1 and dominant language.

### 2.2 Listeners

English listeners (n=44) were very similar to the English control talker group described above. Their ages ranged from 18 to 22 yrs old. These listeners were recruited *via* the Northwestern University Linguistics department subject pool. All English listeners reported acquiring English at birth (age 0) and using English exclusively in the home during childhood in the United States. Usage of English at home and in society was reported at 100% during both childhood and adulthood. Critically, English listeners took no courses in high school in which the medium of instruction was any other language besides English, ensuring that English is both their L1 and dominant language. English listeners with second-language education (e.g., Spanish courses in high school) were included, yet no English listener reported acquiring a second language before age 8.

Similarly, Spanish listeners (n = 33) were very similar to the Spanish control talker group described above and were recruited using similar fliers around Northwestern University's campus. Their ages ranged from 23 to 35 years old. All Spanish listeners reported acquiring Spanish at birth (age 0) and using Spanish exclusively in the home during childhood. Spanish listeners were born in Chile (n = 5), Colombia (n = 6), Mexico (n = 12), Peru (n = 6), Puerto Rico (n = 1), and Venezuela (n = 2). Similarly to the Spanish talkers, Spanish listeners at the time of the study used English rather extensively in their graduate school careers (ranging from 20% to 80% English usage at Northwestern University) compared to their exclusive Spanish usage during childhood (near 100% Spanish usage) and before arrival in the USA for graduate school. Critically, Spanish listeners took no courses in high school in which the medium of instruction was any other language besides Spanish, ensuring that Spanish is both their L1 and dominant language.

No listener in either language group participated in the experiment as a talker.

### 2.3 Stimuli

The stimuli in this experiment consisted of 110 simple sentences in English and Spanish (n = 110 in each language) taken from Soli and Wong (2008) and widely referred to as the Hearing in Noise Test (HINT) sentences. These sentences were chosen because they were specifically adapted for audiometric testing with listeners in their respective native language when presented with additive noise. They have been normed for lexical status of words, grammatical complexity, and sentence length in each respective language and have been used in a variety of cross-linguistic speech intelligibility experiments since their norming. The selection of these 110 sentences (more than 110 exist in English and Spanish) was arbitrary and limited so as not to exhaust participants transcribing more than one hundred sentences in noise.

Talkers were presented with each sentence individually and asked to produce the sentence as naturally and accurately as possible. English and Spanish controls were recorded in their respective L1s while SDB were recorded in both Spanish (their non-dominant L1) and English (their dominant L2). English controls were given course credit for their participation. Spanish controls and SDB talkers were paid $10 an hour for their participation. The recording of these sentences typically took less than 20 min to complete. Talkers were instructed to read each sentence at a normal pace and to repeat any sentence that contained disfluencies (e.g., segmental and/or lexical stress errors, long pauses, word substitutions due to mis-readings). The best production of each sentence was then selected, trimmed to remove silent portions from the ends, and leveled to ensure equal average intensity at play-out [i.e., set to a relative 65 decibels (dB) in praat (Boersma and Weenink, 2020)].

In order to highlight subtle differences in intelligibility of the sentence productions by the three talker groups (SDB, L1-dominant English controls, and L1-dominant Spanish controls), all sentence stimuli were mixed with speech-shaped noise. The noise track was created in praat (Boersma and Weenink, 2020) using a customized praat script (written by the author). In this script, the average long-term average spectrum (LTAS) of each talker was measured across all sentences and then averaged across all talkers to produce a single LTAS-curve. White noise was then generated in praat and then filtered along this LTAS curve creating speech-shaped noise customized for this experiment (i.e., noise that follows the spectral curve of the speech stimuli).

### 2.4 Procedure

English listeners were given course credit for their participation and Spanish listeners received $10 an hour for their participation. Listeners were seated in a sound attenuated booth and presented with the full set of 110 sentences in their respective L1. Sentences were presented in random order over headphones using max/msp software [max 7 (Cycling '74, 2018)]. Listeners were presented with sentences blocked by talker group (e.g., English listeners heard only an entire block of SDB-produced English or English-control produced English, not mixed; the same applies for Spanish). Furthermore, the speech-shaped noise was mixed with each sentence in max/msp at either a –4 dB signal-to-noise ratio (SNR) or a –8 dB SNR, blocked by listener. That is, listeners only heard either controls or SDB at either –4 dB SNR or –8 dB SNR (e.g., some English listener only heard SDB English sentences at –4 dB SNR while some other Spanish listener only heard Spanish-control Spanish sentences at –8 dB SNR). There was a 100 ms delay between sentences with an additional 100 ms lead of noise before the sentence was presented and a 100 ms tail of noise after the sentence ended. In order to minimize any spurious talker-listener pairings that may result in lower speech intelligibility scores (e.g., for some arbitrary reason, talker 1 may be poorly understood by listener 8 but understood well by listener 9), sentences were evenly distributed across talker-listener pairs. To summarize, experimental conditions were blocked by both talker group (L1-dominant English control, L1-dominant Spanish control or SDB) and SNR ( –4 dB SNR or –8 db SNR), but listeners were evenly distributed across all sentence-talker-SNR combinations.

Listeners were instructed to type as many words as possible that they heard over the headphones, even if it was just one word. Listeners were required to type something to move on to the next sentence; as such, responses such as “I am not sure” or “yo no sé” (I do not know) were allowed but then considered wholly incorrect. Participants finished within 30 min. The typed output of each listener was then compared to the correct target sentence and with each word being scored as either wholly correct (receiving a score of “1”) or incorrect (some deviation from the correct form, receiving a score of “0”). Spanish accent marks were ignored and minor spelling errors (i.e., errors that did not result in a different word) were considered “correct.” Scores were then averaged within each sentence (e.g., 5/7 words correct for sentence 103) and then averaged for each talker (total percentage of words correctly recognized across all listeners for a given talker) at each SNR to produce two speech intelligibility scores per talker and per language (SDB only): intelligibility at a relatively easy (–4 dB SNR) and at a relatively difficult SNR (–8 dB SNR). Proportional scores were then log-odd transformed log((p)/(1 − p)) where p is the intelligibility score ranging from 0 to 1 for analysis.

## 3. Results

All data were analyzed using the software r (version 3.1.0, 2014) using linear mixed effects regressions (LMERs) with random intercepts for participant. The factors of interest, signal-to-noise ratio (SNR) and participant group (SDB or L1-dominant talker), were contrast coded (0.5 for “–4 dB SNR” and “control”; –0.5 for “–8 dB SNR” and “switched-dominance bilingual,” respectively) before building any models. The dependent variable (DV) was the log-odd transformed speech intelligibility score log((p)/(1 − p)), where p is the proportional intelligibility score ranging from 0 to 1. These scores were analyzed as log-odds in order to remove the non-linear relationship of incremental changes in proportions (expressed as percent words correctly identified) at various places on the scale (e.g., the difference between 52% and 62% is not equivalent to a difference between 88% to 98%). Significance was assessed using the likelihood ratio test in which the degree to which the data are fit by a full model versus a model excluding a factor (n − 1) or interaction of interest is measured. Final models for both English and Spanish converged with log-odd transformed speech intelligibility as the dependent variable, a two-way interaction, and both main effects for the two factors of interest with random intercepts for participants. Random slopes by SNR were not included as each talker has only one score associated with each level within SNR, causing the model to be over-specified if present.

### 3.1 English results

**Fig. 1.**
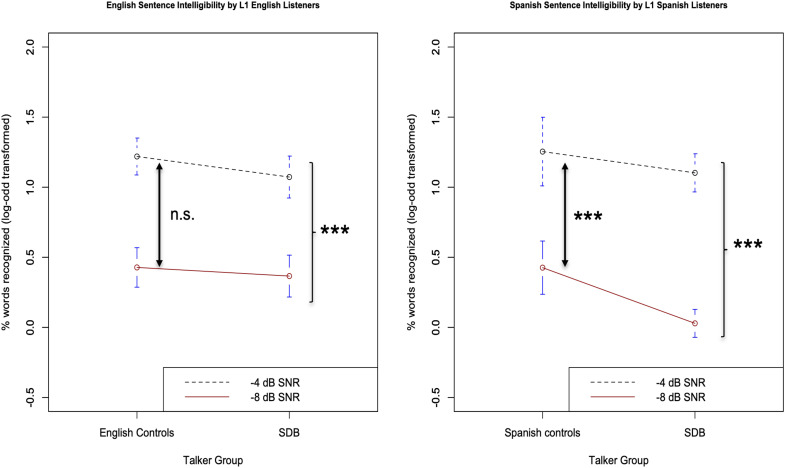
Intelligibility (proportion of words correctly recognized, log-odd transformed) of English (left panel) and Spanish (right panel) speech produced by SDB and L1-dominant control speakers in each language. Error bars represent 95% confidence intervals around the mean.

The average log-odds intelligibility score was 0.43 (s.d. 0.21) in –8 dB SNR and 1.22 (s.d. 0.2) in –4 dB SNR for L1-dominant English controls. The average log-odds intelligibility score was 0.37 (s.d. 0.21) in –8 dB SNR and 1.07 (s.d. 0.21) in –4 dB SNR for SDBs in English. There was a significant main effect of SNR on speech intelligibility scores (β estimate = 0.75, standard error *β* = 0.03, *χ*^2^(1) =  86.97, t = 28.79, p < 0.01), indicating that speech intelligibility scores were significantly lower for –8 dB SNR compared to –4 dB SNR. This is a reliable effect and has been shown in many previous speech-in-noise recognition studies [e.g., Mayo *et al.* (1997); Bradlow and Alexander (2007), and many others]. The main effect of group was not significant (*β* estimate = –0.1, standard error *β* = 0.09, *χ*^2^(1) =  1.56, t = –1.2, p > 0.05), indicating that the speech intelligibility scores for English controls and SDBs were not different. The interaction between group and SNR was also not significant [*β* estimate = –0.08, standard error *β* = 0.05, *χ*^2^(1)  =  2.76, t = –1.63, p = 0.09], indicating that the impact of SNR on English speech intelligibility scores did not differ between groups. These results are shown along with the Spanish results in Fig. 1.

### 3.2 Spanish results

The average log-odds intelligibility score was 0.42 (s.d. 0.27) in –8 dB SNR and 1.25 (s.d. 0.34) in –4 dB SNR for L1-dominant Spanish controls. The average log-odds intelligibility score was 0.03 (s.d. 0.14) in –8 dB SNR and 1.1 (s.d. 0.19) in –4 dB SNR for SDBs in Spanish. There was a significant main effect of signal-to-noise ratio (SNR) on speech intelligibility scores [*β* estimate = 0.95, standard error *β* = 0.05, *χ*^2^(1)  =  67.03, t = 17.47, p < 0.01], indicating that speech intelligibility scores were significantly lower for –8 dB SNR compared to −4 dB SNR. This result is consistent with previous research [e.g., Mayo *et al.* (1997) and many others] as well as the English results above. The main effect of group was also significant (*β* estimate = –0.28, standard error *β* = 0.1, *χ*^2^(1) = 7.51, t = –2.87, p < 0.01), indicating that the speech intelligibility scores for Spanish controls were significantly higher than SDB' Spanish scores. The interaction between group and SNR was also significant (*β* estimate = 0.25, standard error *β* = 0.11, *χ*^2^(1)  = 4.97, t = 2.25, p < 0.05). A *post hoc* comparison revealed that SDB Spanish speech intelligibility scores were more adversely impacted by the more difficult SNR (–8 dB SNR) compared to Spanish controls [unpaired, Bonferroni corrected t-test at –8 dB SNR t(14) = –4.19, p < 0.001; unpaired, Bonferroni-corrected t-test at –4 dB SNR t(14) = –1.22, p > 0.05]. These results are show in Fig. 1 along with the English results.

## 4. Discussion

The aim of this study was to compare the overall intelligibility of speech produced by SDBs in their non-dominant L1 and in their dominant L2. Building on prior work that demonstrated long-lasting benefits of early exposure for segmental production accuracy, we extended the scope of investigation to intelligibility of words in sentences as presented to L1 listeners of each language under both favorable and unfavorable listening conditions.

### 4.1 Discussion of English results

The main question of this subsection is whether the delayed but extended use of language resulting in L2 (rather than L1) language dominance, was sufficient to overcome any differences that may have been initially present in SDBs' delayed onset (but ultimately dominant) L2 speech. SDB did not show speech production differences in their dominant L2 (English) as the range of speech intelligibility scores for SDB was similar to that of English controls. This result is consistent with other studies that have shown L2-dominant speakers (i.e., SDBs) produce native-like speech sounds in their dominant L2 (Au *et al.*, 2002; Godson, 2004; Oh *et al.*, 2003; Yeni-Komshian *et al.*, 2000). The present data establish that this pattern extends to connected speech production in the dominant L2 of SDBs.

### 4.2 Discussion of Spanish results

The main question of this subsection was whether early, but interrupted, acquisition was sufficient to preclude any differences in the language acquisition process of the non-dominant L1 in adult SDBs, specifically with respect to connected speech intelligibility for L1-dominant listeners. In the relatively easy SNR (–4 dB SNR), L1-dominant Spanish listeners showed similar recognition accuracy for words in sentences produced by SDB and Spanish controls. However, the significant main effect of group (L1-dominant Spanish control talkers received higher speech intelligibility scores compared to SDB) and the significant group by SNR interaction (SDB speech intelligibility was more adversely affected by the more difficult –8 dB SNR compared to that of Spanish controls), suggests that the interrupted L1 acquisition and replacement by a dominant L2 resulted in reduced resistance to signal degradation by high levels of background noise, at least for L1-dominant listeners.

It may appear that these results conflict with Au *et al.* (2002) and Oh *et al.* (2003), which have shown dominant L1-like production of speech sounds in the non-dominant L1 of SDBs. However, one difference between these studies and the current study is that we assess both the (non-dominant) L1 and (dominant) L2 of SDBs compared to the L1 of L1-dominant talkers. Au *et al.* (2002) and Oh *et al.* (2003) assess the (non-dominant) L2 of late learners compared to the (non-dominant) L1 of SDBs. Therefore, both sets of results are possible: non-dominant L1 speech production by SDBs may be distinct from both non-dominant L2 production of late learners and dominant L1 speech production. These relatively fine-grained differences underscore the distinct influences of timing (relatively early versus relatively late) and duration (limited versus extended) of exposure during speech and language learning. Additionally, in the –4 dB SNR (i.e., “easier”) condition of the present study, SDBs' sentence productions in their non-dominant L1 Spanish received comparable recognition accuracy scores to those of Spanish controls, consistent with Au *et al.* (2002) and Oh *et al.* (2003) that have demonstrated, in some cases, dominant L1-like speech production in the non-dominant L1. Critically, it was only in the context of high levels of background noise that differences arose between dominant L1 and non-dominant L1 speakers. It is important to note that the reduced word recognition accuracy for SDB Spanish in the −8 dB SNR condition involved L1-dominant Spanish listeners. It remains an open question whether SDB listeners drawn from the same population as the talkers (i.e., L2-English dominant listeners) would show a difference in SDB Spanish speech production due to their shared speech and language experiences.

## 5. Conclusion

The goal of the current study was to determine how adult speech production reflects the timing (i.e., age of acquisition) and amount (i.e., length of acquisition) of language exposure. The SDBs in this study provided unique insight into the subtle differences in language function not readily available in monolingual or bilingual speakers for whom the first-acquired language (L1) is also the dominant language. Results showed that, for L1-dominant English listeners, intelligibility of dominant L2 English as produced by SDB Spanish speakers was indistinguishable from that of L1-dominant English speakers even under a highly unfavorable (–8 dB) signal-to-noise ratio condition. This indicates that, with respect to speech intelligibility, extended exposure can successfully provide benefit even with delayed exposure to the L2. However, the converse does not appear to hold. That is, early exposure to the L1 does not entirely guard against subtle speech production differences associated with interrupted exposure to the L1. Specifically, the data showed substantially lower intelligibility of non-dominant L1 Spanish for L1-dominant Spanish listeners under a highly unfavorable (–8 dB) signal-to-noise ratio listening condition. These results suggest that with respect to speech production learning, while early exposure has some benefits, extended exposure can compensate for later onset of acquisition.
